# The Need for Consumer-Focused Household Food Waste Reduction Policies Using Dietary Patterns and Socioeconomic Status as Predictors: A Study on Wheat Bread Waste in Shiraz, Iran

**DOI:** 10.3390/foods11182886

**Published:** 2022-09-17

**Authors:** Shahin Ghaziani, Delaram Ghodsi, Karsten Schweikert, Gholamreza Dehbozorgi, Hamid Rasekhi, Shiva Faghih, Reiner Doluschitz

**Affiliations:** 1 Department of Computer Applications and Business Management in Agriculture, University of Hohenheim (410C), 70593 Stuttgart, Germany; 2 Department of Nutrition Research, National Nutrition and Food Technology Research Institute, Faculty of Nutrition Sciences and Food Technology, Shahid Beheshti University of Medical Sciences, Tehran 19816-19573, Iran; 3 Core Facility Hohenheim (CFH), University of Hohenheim (640), 70593 Stuttgart, Germany; 4 Horizon Smart SAT (Surveillance and Analysis Technology), Fars Science and Technology Park, Shiraz 71976-87811, Iran; 5 Department of Community Nutrition, School of Nutrition and Food Sciences, Shiraz University of Medical Sciences, Shiraz 71348-14336, Iran; 6 Nutrition Research Center, Shiraz University of Medical Sciences, Shiraz 71348-14336, Iran

**Keywords:** household food waste, waste related behavior, sustainable consumption, regression model, food waste occurrence

## Abstract

Current household food waste (HFW) reduction plans usually focus on raising consumer awareness, which is essential but insufficient because HFW is predominantly attributed to unconscious behavioral factors that vary across consumer groups. Therefore, identifying such factors is crucial for predicting HFW levels and establishing effective plans. This study explored the role of dietary patterns (DP) and socioeconomic status (SES) as predictors of HBW using linear and non-linear regression models. Questionnaire interviews were performed in 419 households in Shiraz during 2019. A multilayer sampling procedure including stratification, clustering, and systematic sampling was used. Three main DPs, i.e., unhealthy, Mediterranean, and traditional, were identified using a food frequency questionnaire. Results indicated that a one-unit rise in the household’s unhealthy DP score was associated with an average increase in HBW of 0.40%. Similarly, a one-unit increase in the unhealthy DP score and the SES score increased the relative likelihood of bread waste occurrence by 25.6% and 14.5%, respectively. The comparison of findings revealed inconsistencies in HFW data, and therefore the necessity of studying HFW links to factors such as diet and SES. Further investigations that explore HFW associations with household characteristics and behavioral factors will help establish contextual and effective consumer-focused plans.

## 1. Introduction

Food loss and waste (FLW) occur at different stages of agri-food supply chains, including the reduction in food mass along the production, postharvest, processing, and distribution stages, terminologically referred to as ‘food loss’ [1], as well as food discard at retail, foodservice, and household levels, generally defined as ‘food waste’ [2]. Throughout this paper, the terms ‘food loss’ and ‘food waste’ are used in accordance with the abovementioned definitions. Based on the latest assessments, 14% of food is lost in upstream food supply chains [1], and 17% is wasted at the consumption level [2].

From a lifecycle perspective, the food waste that occurs at the final stages of food supply chains, especially in households, may cause a higher economic and environmental impact than food loss at earlier stages [1]. The food that reaches a household has the footprint of the retail stage in addition to the upstream supply chain, and when it is wasted, the impacts of cooking and domestic storage are also added. In the meantime, the amount of household food waste (HFW) is enormous. The HFW accounts for up to 36% of the total FLW and 65% of food waste [2]. However, it appears that the strategies for reducing HFW are rather general and unfocused, in contrast to the specific plans for tackling the FLW in industrial and business agri-food sectors, including retail and foodservice.

For example, the United States created the FLW 2030 champions initiative in 2016, aiming to halve FLW by 2030 by engaging businesses in food production, processing, retail, and foodservice, but it did not address HFW [3]. In 2019, Germany initiated the national strategy for food waste reduction, which involved all food supply chain sectors, but adopted a general approach toward household consumers [4]. Similar examples can be found in other countries, most of which, at best, proceeded as far as providing general guidelines for reducing HFW [5,6,7]. HFW reduction guidelines help raise awareness and consciousness with regard to waste generation. However, the role of consumers’ conscious intention to reduce food waste is not as determinative as the role of food-related behavior and habits [8]. Moreover, a major HFW data gap exists in developing countries [9]; meanwhile, many of these countries tend to follow the same strategies formulated for developed countries, which does not necessarily lead to desirable outcomes. Even though guidelines can be effective to some extent, the significance of specific plans tailored for different types of consumers in specific sociogeographical settings cannot be emphasized enough [10]. Such plans scarcely exist.

In the United Kingdom, the action on food waste launched by the waste and resources action programme (WRAP) in 2000 has resulted in tremendous progress toward studying and reducing FLW along food supply chains [11]. In 2014, WRAP published a report that shed light on the association of HFW with household characteristics, e.g., sociodemographic and food-related behaviors [12]. Accordingly, WRAP initiated the “Love Food Hate Waste” campaign, targeting 18 to 35 year-old age groups [13]. However, additional knowledge about HFW attributions is still required to effectively establish further consumer-focused plans.

Determining the household characteristics associated with HFW could also facilitate finding predictors for estimating HFW levels. The HFW quantification methods are either too costly and labor-intensive, i.e., direct measurement and waste composition analysis, or too inaccurate, i.e., recall questionnaire and diary recording [14,15,16,17,18,19,20]. The HFW predictors could enable researchers and decision-makers to evaluate the level or occurrence of food waste in households without the complications of waste quantification.

Food is wasted in households for various reasons, such as consumers’ gustatory preferences [21], food purchasing and storing [22,23], beliefs and concerns, and food preparation [24,25,26,27]. Nonetheless, the question of how the dietary pattern (DP), which plays a central role in consumption behavior, affects the HFW has not yet been established. The USDA defines DP as “the quantities, proportions, variety, or combination of different foods, drinks, and nutrients (when available) in diets, and the frequency with which they are habitually consumed.” ([28] p. 9).

Consumers may not be well aware of the environmental and economic impacts of their daily food choices [29,30]. However, the habitual food consumption that constitutes the DPs cumulatively imposes an enormous impact on the environment and the economy in different ways, including through food waste generation [31,32,33,34,35,36,37]. Some researchers acknowledged diet as a factor linked with HFW [12,38,39,40,41,42,43,44,45,46]. However, to the best of our knowledge, no study specifically focused on how HFW is associated with DP.

This paper aimed to investigate whether HFW is associated with DPs. Because the economic situation can impact food waste levels [47,48,49], the effect of socioeconomic status (SES) on HFW has also been analyzed. Considering the existing food waste data gap at the household level in developing countries [2,9,50], Iran was chosen as an example to conduct the research. This study focused on household wheat bread waste (HBW) in Shiraz, Iran. Wheat bread, hereafter referred to as bread, was chosen because it is the main staple food in the country [51]. Bread is one of the 14 main food items in the Iranians’ basic food basket [52], and its average daily intake is known to be 320 g per capita [53]. Shiraz is the capital of Fars, the major wheat-producing province of Iran.

## 2. Materials and Methods

### 2.1. Study Design

The survey was performed from December 2018 to August 2019 in Shiraz, Iran. A total of 419 households were interviewed by a group of 13 trained assistants. A household was defined as two or more residents of one house sharing food and its costs. Preferably the mother or the wife was selected as the interviewee because their dietary intake reportedly mirrors the nutritional status of other family members [45,54,55,56,57]. If they were unavailable, the person who is usually in charge of the household’s food shopping and preparation was interviewed. A three-stage sampling approach was employed, consisting of stratification, clustering, and systematic sampling. The sample size determination and the sampling procedure are thoroughly described by Ghaziani et al. [58].

### 2.2. Questionnaire

A questionnaire was designed to obtain information on the household level in three sections: (1) demographics and SES, (2) dietary intake, and (3) bread purchasing and wastage. The questionnaire was tested beforehand by conducting 22 interviews with randomly selected households outside the study population to ensure adequate comprehensibility of the questions. The questionnaire sections are described below.

#### 2.2.1. Demographic and Socioeconomic Section

The demographic and socioeconomic questions addressed the household size, income, housing characteristics, house ownership status, and the head of household’s occupation and education level. Moreover, binary questions about the ownership of 11 durable assets were asked.

#### 2.2.2. Dietary Section

A 168-item semi-quantitative food frequency questionnaire validated by Esfahani et al. [59] was employed to gather dietary intake data. The questionnaire required the interviewees to report estimations of the intake of each food item, on a daily, weekly, monthly, or yearly basis, within a maximum of a one-year span.

#### 2.2.3. Bread Waste Section

The HBW quantification was performed using a self-assessment approach by means of a recall questionnaire. The focus was on ten commonly consumed bread types, identified according to the Iranian National Standardization Organization [60,61], consisting of two main categories. i.e., traditional bread (TB) and non-traditional bread (NTB). Detailed specifications of the bread types and the HBW amount quantification method are described by Ghaziani et al. [58].

### 2.3. Statistical Analysis

IBM SPSS Statistics version 25 [62] was used to analyze the data, with a significance level of *p* < 0.05. The socioeconomic, dietary, and HBW data analyses are explained below.

#### 2.3.1. Socioeconomic Data Analysis

The socioeconomic data were analyzed based on the method explained by Vyas and Kumaranayake [63] by applying principal component analysis (PCA) to the socioeconomic variables. The factor scores of the principal component (PC) with the highest eigenvalue of 4.12, explaining 18.75% of the variance in data, were selected as weights of the SES indicator variables. The SES score for each household was computed according to the equation below. Higher scores represent households with higher SES. For a simpler description of the data, the households were grouped by assigning cut-off values for percentiles of the study populations. The percentiles were set according to Filmer and Pritchett [64], identifying the lowest 40% as poor, the next 40% as middle class, and the top 20% as rich.

$$y_{i}=\overset{22}{\underset{n\;=\;1}{\sum }}x_{n}C_{n}$$

where *y* is the SES score, *i* is the household’s number (with *i* = 1 to 419), *x_n_* is the household’s value for the *n*th SES indicator, and *C_n_* is the PC load of the *n*th SES indicator.

#### 2.3.2. Dietary Data Analysis

The participant’s total intake of the 168 food items of the FFQ was separately converted to gram intake per day. The food items were merged into 30 categories based on their nutrient content, researchers’ opinions, and the study of Hosseyni Esfahani et al. [65], presented in Table 1. Each participant’s total daily intake of different food categories was calculated by totaling daily intakes of their corresponding food items. PCA was applied to find the main components responsible for most of the variance in data, assigning the food categories as variables.

The adequacy of the correlation matrix of the predefined food categories for PCA was examined using the Kaiser–Meyer–Olkin Measure (KMO) test. The test showed a significant result with a *p*-value lower than 0.001 and a KMO value of 0.708, indicating acceptable adequacy for conducting PCA [66].

Based on the initial results and visual inspection of the scree plot, three components with the highest eigenvalue (4.025, 1.914, 1.747), explaining 25.64% of the variance, were identified for extraction. The rotation method was set on Varimax with Kaiser normalization. Coefficient factors below the minimum absolute value of 0.2 were suppressed, and other values were used to identify the food categories with primary loads in each component. Ultimately, three main DPs were identified based on the nutritional interpretability of food categories loaded together within each component and according to Mirmiran et al. [67]. Each household received a score for each DP, which was calculated according to the equation below. The mean score values of each DP were compared across the SES classifications using Least Significant Difference (LSD) tests.

$$y_{ij}=\overset{30}{\underset{n=1}{\sum }}x_{n}L_{ni}$$

where *y* is the DP score, *i* is the component number representing each DP (with *i* = 1 to 3), *j* is the household’s number (with *j* = 1 to 419), *x_n_* is the daily intake of the *n*th food category, and *L_ni_* is the load of the *n*th food category within the *i*th DP.

#### 2.3.3. Bread Waste Data Analysis

The waste mean values for each bread type were calculated as described by Ghaziani et al. [58]. The mean waste value for TB and NTB were calculated as the average of the waste amounts of all bread types within their respective category. Paired samples and independent samples t-tests were implemented to compare the mean waste across bread categories.

#### 2.3.4. Regression Models

The HBW amount relationship with the DP and SES scores was analyzed using multiple linear regression by assigning the waste amount as the dependent variable and the three DP scores and the SES score as regressors. Additionally, a binary logistic regression model analyzed the occurrence/non-occurrence of bread wastage depending on the variation in the DP and SES scores. Moreover, consuming or not consuming NTB in relation to DP and SES scores was explored using binary logistic regression.

## 3. Results

### 3.1. Demographics and Socioeconomic Status

A total of 1548 people lived in the studied households, with an average household size of 3.69 (SD = 1.22). Table 2 indicates which members were interviewed, responsible for household nutrition and heads of the households. As intended, mothers who are most often responsible for food preparation were mainly interviewed. The majority of households were male-headed. The table also shows the proportion of different occupations and education levels among the heads of households. Moreover, the proportion of different SES classes is presented.

### 3.2. Dietary Patterns

Table 3 shows details regarding the load of food categories on each component. According to nutritional interpretation of the components, three DPs were identified, with component 1 being unhealthy, 2 Mediterranean, and 3 traditional. The household score for each DP indicates their tendency to implement that DP habitually.

Table 4 presents how the DP mean scores vary across SES classes. The LSD test suggests that the unhealthy mean score was higher in the rich class. Additionally, the Mediterranean scores were significantly higher in richer SES classes. A reverse outcome was observed in the traditional scores, with the richest class scoring lowest.

### 3.3. Bread Waste

Three respondents did not answer the HBW questions (0.72% missing). The total average HBW was 1.80% (n = 416, SD = 3.36). The mean waste values were 1.70% (n = 416, SD = 3.70) and 2.50% (n = 304, SD = 5.26) for TB and NTB, respectively. The paired sample t-test did not indicate significant differences between the two bread categories. Because the paired comparison excluded the non-NTB-consumers, an independent samples t-test was employed, revealing that the NTB waste was significantly higher than the TB waste (*p* = 0.016).

**Figure 1:**
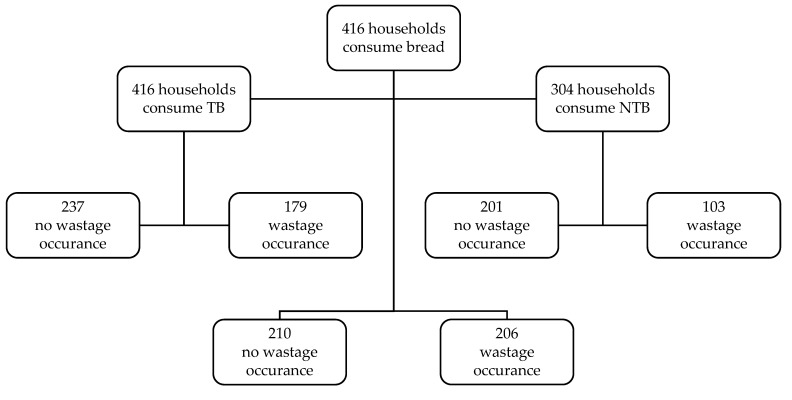
Schematic presentation of the number of households that consume bread and the number of households where bread wastage occurs, grouped based on bread categories; TB = traditional bread, NTB = non-traditional bread.

Figure 1 illustrates the frequency of bread consumption and wastage occurrence in the studied households based on bread categories. Out of 416 respondents, 50.48% reported that they do not waste bread. All 416 households consume at least one type of TB, among which 56.97% reportedly did not generate any TB waste. A total of 73.08% of the households consume NTB, of which 66.12% claimed that the NTB is not wasted in their households.

### 3.4. The Effects of the Households’ Dietary Patterns and Socioeconomic Status on Bread Waste

Table 5 presents the results of multiple linear regression models for predicting the HBW amount by the variation in the DP and SES scores. The models revealed that the unhealthy DP had a significant positive influence on the waste amount. This could mean that, for a one-unit increase in the unhealthy DP score, the HBW amount increases by 0.40% on average, supposing that other variables are constant. The Mediterranean and traditional DPs were insignificant in the regression models. The regression model for TB waste amount detected a marginally significant coefficient for the unhealthy score, implying that the unhealthy diet score variation might be able to predict the TB waste amount. The NTB waste was not affected by any DPs. Moreover, the effect of SES on the HBW amount of all categories was insignificant.

Because not all households consume NTB, a binary logistic regression model was employed to assess the predictability of the NTB consumption when the DP and SES scores vary (see Table 6). The outcome revealed that by a one-unit rise in a household’s unhealthy and Mediterranean scores, the relative probability of consuming NTB increases by 79.3% and 49.6%, respectively. Consuming NTB could not be predicted by the traditional DP and SES.

As most of the factors in linear regression could not predict the HBW amount, binary logistic regression models were applied to examine whether the variation in the variables could predict the wastage occurrence. Table 7 shows the likelihood of bread wastage by variation in DP and SES. In line with the multiple linear regression results for the waste amount, the unhealthy diet positively impacted bread wastage, meaning that raising the unhealthy diet score by one unit would increase the relative probability of wastage occurrence by 25.6%. Meanwhile, the effect of Mediterranean and traditional DPs remained insignificant. Additionally, no DP impacted the TB and NTB wastage.

The SES score significantly explained the wastage occurrence such that a one-unit rise in the SES score would increase the relative odds of wastage by 14.5%. The impact of the SES on the TB wastage was marginally significant (11.3% relative chance of wastage per one-unit SES increase), and on NTB wastage was significant (a one-unit rise in the SES score would increase the relative odds of the NTB wastage by 23.2%).

## 4. Discussion

The current study revealed that HBW could be influenced by DP and SES. The effects of DP and SES on HBW were assessed using two types of models, i.e., multiple linear regression and binary logistic regression. The first model examines the predictability of waste amount depending on the variation in the main factors, namely, DPs and SES. The latter predicts the relative probability of HBW occurrence if the regressors’ values change. The linear regression outcome revealed that explanatory variables other than unhealthy DP do not influence the BW amount. This outcome could be a result of limited waste values or small NTB subsamples in the dataset from this study. The reasons for obtaining such limited waste amounts are thoroughly discussed by Ghaziani et al. [58]. These reasons may include inconsistencies in food waste conceptual and methodological frameworks, change in domestic food storage methods, the unprecedented economic recession in Iran, bread quality improvement, and cultural stigmatization of bread wastage [58]. More than half of the respondents reported that they do not waste bread (zero-wasters). Because the linear regression model only indicated a significant effect of one regressor on the waste amount, a binary logistic regression model was employed to detect possible influences of other variables on bread wastage occurrence, as measuring the bread wastage occurrence or non-occurrence is less error-prone than measuring the exact waste amount.

Both the amount and occurrence of HBW were positively influenced by unhealthy DP, meaning households with higher unhealthy DP scores were likely to waste more bread, and bread wastage was more likely to occur in their houses. Moreover, between the two bread categories, TB waste could be positively affected by the unhealthy DP score, as the coefficient was marginally significant. The Mediterranean and traditional DPs and SES played no role in the variation in the HBW amount, regardless of bread categories. However, the binary logistic regression model detected a significant effect of SES on HBW occurrence, meaning that bread wastage was more likely to occur in households with higher SES.

In general, some studies confirmed that HFW could be associated with diet and eating habits without investigating the direct relationship between diets and HFW [8,39,40,44,68,69]. Other researchers have acknowledged food choices and shopping preferences as diet-related factors influencing HFW [40,70]. Two studies analyzed the change in HFW in relation to diet [45,46]. Conrad et al. [46] found that higher diet quality is associated with higher HFW in the United States using linear regression models, but HBW (grains and mixed grain dishes) was not significantly influenced. In a similar study in Canada, Carroll et al. [45] only found that daily fruit and vegetable waste amount in households was positively associated with the parents’ diet quality, while the diet quality effect on the waste amount in other food groups, including bread, was insignificant.

In this study, the diet quality was not analyzed, and other food groups such as fruits and vegetables were not included either. The reason for choosing DP over diet quality was that DP is more identifiable as a predictive factor for designing food waste reduction policies and intervention programs. Bread was chosen due to its high importance (see Section 1), while other food groups were not included due to logistical constraints. However, if the households with higher unhealthy DP scores are assumed to have a lower diet quality based on their predominant food choices (see Table 3), the findings from the present study could be compared to the ones of Carroll et al. [45] and Conrad et al. [46]. Given this assumption, the current results contradict Carroll et al. [45] and Conrad et al.’s [46] findings regarding the link between HBW and dietary quality.

However, a common finding in Carroll et al. [45] and Conrad et al.’s [46] studies and the current one was that food choice could be a major factor affecting HFW. The present study revealed that the NTB mean waste value was higher than the TB mean waste. Meanwhile, the households with higher unhealthy DP scores were more likely to consume NTB. The evidence suggests that mismanagement in shopping for and preparation of perishable foods would lead to a high level of HFW [40]. Based on anecdotal evidence, the NTB in Shiraz is usually sold in packed units, kept at room temperature, and consumed fresh. Meanwhile, TB bread is normally purchased as pieces and stored in a freezer [58]. Failing to consume the whole package is a reason for food wastage, especially in smaller households [22]. Furthermore, many consumers in Shiraz discard the inner crumbs of some NTB types, such as baguette or hamburger bun, which can be why NTB waste is higher than TB [17]. Ergo, one reason for wasting more bread in households with higher unhealthy diet scores could be that their choice of bread involves potentially higher waste generation. Interestingly, Conrad et al. [46] and Carroll et al. [45] argued that the higher HFW amount in the households with higher diet quality is basically due to higher consumption of fruits and vegetables, which perish more rapidly than most food groups. Therefore, food choice was evidently an influential factor for HFW in all three studies.

The general inadvertency toward food consumption in consumers with higher unhealthy DP scores could offer a potential explanation for their higher HBW amount in the present study. Consumers with a high tendency toward unhealthy diets have a relatively low level of consciousness about their health and food consumption behaviors [71,72]. Parizeau et al. [41] found that the households with a member who has a special diet, such as vegetarian or diabetic, have more consciousness about their food consumption and tend to adopt HFW reduction strategies. Consumers concerned about sustainable and healthy food consumption are more willing to reduce or reuse food waste [73]. On the other hand, consumers’ lack of concern for their food-related behavior may cause them to not have the intention to restrict HFW [8]. Of course, this may not be the case in certain circumstances, as consumers with higher diet quality generated more HFW in the United States and Canada [45,46]. This disparity could be due to differences in other HFW-relevant aspects, such as religious, cultural, psychographic, and socioeconomic factors [73,74,75]. Therefore, using a single aspect to compare HFW results may reveal contradictions.

For example, dietary habits vary strongly based on psychographics and cultural factors [76,77,78,79,80,81]. The dichotomy between the present findings and the studies in the United States and Canada [45,46] regarding HBW’s link to SES also exists in the link between SES and diet across the two geographical regions. The current results indicated that the average unhealthy diet score was lower in the bottom socioeconomic classes. This is in good agreement with another study in Iran by Abdollahi et al. [82]. Meanwhile, evidence suggests that North American households with higher SES tend to adopt healthier dietary habits [83,84,85,86].

Other inconsistencies exist among the findings regarding HFW’s link to SES. Other studies support the present findings that bread wastage is more likely in households with higher SES [87,88,89,90]. On the contrary, a study in Brazil showed that low SES consumer groups generated more HFW due to poor food purchasing and preparation management despite their willingness to cut expenses by consuming food frugally. In another study in Germany, Herzberg et al. [22] found that socio-demographic variables did not influence the HFW amount. All in all, the dynamic between HFW and other factors such as DP and SES highly varies across consumer groups with different demographic and socio-cultural backgrounds. Therefore, possible explanations for such dynamics must be assessed based on the specific circumstances of the target populations.

The positive relationship between HBW and SES in the present study could be attributed to the poorer households’ overall financial circumstances regarding bread consumption. It has previously been reported that bread has a higher share in the composition of the family food basket among Iranian households in the lower socioeconomic classes [52,91]. Such households have a lower purchasing power and, therefore, tend to avoid over-purchasing [47,49] while utilizing their food frugally [92,93]. A study from Greece revealed that households with financial hardships could reduce HFW to restrict their spending [94]. For instance, financial constraints drive consumers to consume food products in suboptimal conditions, which leads to more HFW avoidance [95]. These explanations imply that in the current study, zero-wasters were mostly the poorer households who intended to cut expenses by efficiently utilizing their food resources, with bread being the most important. Nevertheless, a study on HFW in Iran showed that households with higher SES have a higher intention to reduce HFW [96]. This contrast attests to the precedence of behavioral factors over the conscious intention to avoid HFW.

Food-related behaviors are often automated and unconscious [97]. Consumers may not even specifically realize the actual reasons for wasting food [22]. Many consumers have no clear awareness of the HFW quantity or even its occurrence [18,98]. Generally, wasting food is stigmatized in most cultures [99,100,101], and common sense confirms that it has no economic justification. Most consumers are concerned, at least to some extent, about the HFW issue [94,102]. Therefore, it seems that everyone could agree on the necessity of avoiding HFW.

A recent qualitative study involving 23 Chinese household interviews revealed that consumers’ psychological consciousness and religious beliefs could lead to HFW minimization [103]. However, the conscious intention to avoid HFW is not sufficient motivation to avoid HFW. For example, the religious teachings of Islam abominate wasting food [104]. Nevertheless, as a famously religious country, Saudi Arabia ranks fifth globally in terms of HFW, with 105 kg per person annually [105]. In many Muslim countries, substantial food amounts are wasted during religious occasions such as Ramadan [106]. Aktas et al. [107] stated that the high level of food waste during Ramadan is mainly due to changes in food consumption behavior.

Overall, the HFW cannot be attributed to one or two factors, and the number and the types of factors and their impact on HFW differ depending on geographical, demographical, and cultural settings. Households may be the most complicated FLW hotspots along food supply and consumption chains due to the multifaceted nature of food consumption behaviors [108]. It is worth reiterating that most of the existing HFW prevention guidelines are rather general and aimed at increasing consumers’ awareness of the topic [3,4,5,6,7]. As stated in Section 1, most existing guidelines are based on studies and data from developed countries, while a major data gap exists in the developing world [9]. Raising awareness on the issue of HFW is essential but insufficient for achieving satisfactory reduction scales [109].

Imitating general guidelines that are formulated based on limited data from specific sociogeographical regions (mostly developed countries) would not necessarily result in satisfactory outcomes elsewhere. Besides gaining a proper understanding of HFW and its drives, different regions have to set commensurate objectives to be able to strategize efficient FLW reduction. For example, in developed countries with high food security levels, the focus will likely be more on the environmental aspects of FLW by moderating the surplus supply, while less-developed countries may need to focus on improving food security through reusing FLW to feed vulnerable groups [1]. Therefore, the need for devising consumer-focused HFW reduction strategies for each target population cannot be stressed enough. For developing an effective consumer-focused HFW reduction strategy, three steps are essential, namely:

1. finding the factors that affect HFW level and generation and identifying how they make an impact;
2. grouping consumers based on HFW-related characteristics;
3. formulating strategies and policies for HFW reduction focused on behavioral change.

Discovering the behaviors linked to HFW is the key to finding consumer-focused waste prevention strategies. In a systematic review, Schanes et al. [110] categorized the behavioral practices associated with HFW into eight groups, including:

1. planning (i.e., meal planning and checking food inventories before shopping);
2. shopping;
3. storage;
4. cooking;
5. eating;
6. managing leftovers;
7. assessing edibility;
8. disposal.

Nonetheless, more waste-related factors and simpler household characteristics must be identified to act as HFW predictors. Examples of such indicators may include DP, SES, and household age and size. Studies suggested that younger households waste more food than older households [25,69,98,111], or HFW amount is higher in larger households than in smaller ones [18,22,41,88,112,113]. Implementing HFW predictors may facilitate creating proper incentives for avoiding HFW among specific consumer groups.

Because a combination of factors explains the HFW [70], household consumers can be grouped based on multiple waste-related characteristics. Grouping household consumers based on such characteristics can benefit the decision-makers in two ways: first, by using the factors as predictors for estimating the quantity and quality of HFW in different segments of a population; and second, by identifying which factors are most relevant to focus on for formulating consumer-focused HFW reduction plans. Grouping consumers must be based on conveniently measurable factors to facilitate the implementation of the plans. For example, in the American and Canadian studies [45,46], diet quality was used to predict HFW levels, which requires comprehensive data collection. Dietary data in the present study was collected using a food frequency questionnaire, which is also a time and labor-intensive method. However, proxy yet desirable results can be obtained from simpler methods such as food screening [114,115] or simpler questionnaires [116] to project HFW-related factors such as diet quality or dietary patterns. Ultimately, tools and methods for implementing each strategy development step must be chosen according to the available resources.

In the last instance, HFW reduction policies and strategies can be designed for each consumer group, focusing on behavioral change while raising awareness about the HFW issue in parallel. Visschers et al. [111] suggested that food waste reduction programs should target consumers’ behavior in order to gain better results, which seems to be a reliable approach. Zamri et al. [117] recommended that future food waste reduction campaigns should focus on faith to encourage behavioral changes. Moreover, incentives for food waste reductions can simultaneously focus on other beneficial behavioral aspects, such as improving dietary health [45]. Nevertheless, governmental control may lead to lowering consumers’ intention to reduce food waste [118]. Therefore, authorities must take a motivating approach rather than imposing certain policies on the community. Generally, depending on the objectives of a food waste reduction plan and how it is implemented, its direct or indirect impact on the food and nutrition security for different groups of people may vary, and not everyone reaps the benefits [119]. Hence, achieving desired objectives will require thorough assessments of the effects and consequences of each strategy.

As an example, based on the current results, the authorities in Shiraz could assume that bread is probably wasted more in richer districts, particularly among the households with a high tendency toward unhealthy diets and possibly due to higher consumption of NTB. One approach would be to promote waste-reducing actions such as supplying non-packaged NTB in these regions. Moreover, it has been suggested that HFW reduction strategies with multiple objectives that overlap environmental, economic, and social aspects could result in optimal accomplishments [120]. Therefore, implementing a factor such as DP would provide the opportunity to focus not only on waste but also on the health aspects of consumption behavior. For example, encouraging shifting unhealthy diets to healthier alternatives may reduce HBW in Shiraz while improving the communities’ dietary health.

## 5. Conclusions

HFW has become a dilemma with adverse environmental, social, and economic impacts. Although most consumers are unwilling to waste food at home, the HFW still accounts for a substantial share of the total FLW. The amount and occurrence of HFW are attributed to multiple unconscious behavioral factors and household characteristics. Summing up the results, it can be concluded that the HBW amount in Shiraz is associated with DP and its occurrence varies depending on the households’ SES. The inconsistency between the findings presented in this paper with other studies emphasizes the need for developing HFW reduction strategies tailored to specific consumer groups based on their HFW-related characteristics. This outcome could widen the current knowledge of HFW and provide further insight to decision-makers to plan better for reducing HFW.

Nonetheless, a limitation of the current study was that the focus was only on one food commodity, mainly due to limitations in time and research resources. However, the overall findings of the present study corroborate previous results that HFW is, in fact, linked with diet. Additionally, the silver lining of the specificity of this study was that focusing on one food commodity can enable a deeper insight and an exclusive evaluation. Moreover, to the best of our knowledge, this study is the only investigation specifically focused on the relation between HFW and DP based on detailed primary nutritional data. The evidence from this study could persuade the decision-makers and researchers to advert their focus on DP and SES as predictors along with other factors influencing HFW.

Further studies should concentrate on specific food commodities in distinct regions to facilitate the selection of effective objectives and strategies for HFW reduction plans in the respective settings. In this framework, discovering the behavioral aspects and household characteristics that affect HFW is the key. Therefore, further research should focus on more food groups and commodities, additional HFW behavioral drives, and more precise HFW quantification methods. It is essential to assess the HFW issue from multiple perspectives and establish solutions specific to different cultural and geographical settings. Meanwhile, long-term success rests upon reevaluating the HFW and its affecting factors anew as circumstances change, and accordingly, the adjustment of the objectives and strategies have to be taken into consideration.

## Figures and Tables

**Table 1:** Food grouping used in principle component analysis of the 168 food items in the food frequency questionnaire for the identification of dietary patterns.

Food Category	Food Item
Processed meat	Sausages
Red meat	Lamb, beef, veal, minced meat, hamburger
Lamp/veal organ meat	Tripe, heart, liver, kidney, head, feet, tongue, brain
Fish	All fish, fresh or canned
Poultry	All chicken parts
Eggs	Eggs
Hydrogenated fat with animal origin	Cream, butter, tallow, animal fat
Coffee	Coffee
Tea	Tea
Fruits and fruit juice	Apple, apricot, banana, cantaloupe, cherries, citrus juice, dates, fresh fig, fresh fruits and vegetable juice, grapefruit, grapes, greengage, kiwi, lemon juice, vinegar, and verjuice, lime, mulberry, orange, peach, pear, Persian melon, persimmon, plum, pomegranate, strawberry, sweet lemon, tangerine, watermelon
Refined grains	White bread (lavash, baguette, bun, broetchen (bread rolls), mini baguette, toast), rice, pasta/spaghetti, noodles/vermicelli, wheat flour
Whole grains	Wheat whole grain bread (sangak, taftoon, barbari), other whole grain bread types, barley, oatmeal
Legumes	Beans, chickpea, lentil, mung bean, soybean meal, split pea
Low-fat dairy products	Low-fat and skimmed milk, low-fat yogurt, kashk, yogurt drink (doogh)
High-fat dairy products	High-fat, whole and chocolate milk, cheese, high-fat yogurt (incl. concentrated and creamy), ice cream
Margarine and vegetable hydrogenate fat	Margarine, vegetable hydrogenated fat
Other vegetables	Bell pepper, carrot, chili pepper, cooked and raw celery, cooked and raw tomato, cooked green bean, cooked green pea, cooked mushroom, cooked spinach, cucumber, fresh herbs, lettuce, pumpkin, raw and cooked leafy vegetables, raw and fried onion, raw garlic, tomato paste, turnip, zucchini or eggplant
Potato	Baked potato
Salty snacks	French fries, puffs, potato chips, salty crackers
Cruciferous vegetables	Red and white cabbage, other kinds of cabbage
Olive	Olive, olive oil
Pickle	Pickles, salted vegetables
Dried fruits	Dried mulberry, raisin, others (dried fig, follicle, etc.)
Oil	Vegetable oils (except olive)
Nuts	Almond, peanut, pistachio, seeds, walnut
Sweets and desserts	Biscuits, candy, chocolate, gaz, honey and jam, noghl, pastries (non-crème and creamy), sohan, sponge cake, cookies other cakes, sugar, sugar candy, toffy
Sugary beverages	All soft drinks and industrial sugar sweetened beverages
Mayonnaise	Mayonnaise
Diet coke	Diet coke

**Table 2:** Demographic and socioeconomic summary of the studied households (n = 419).

Variables		Frequency	
		*n*	*%*
Respondent	Mother	376	89.7
	Father	14	3.3
	Daughter	22	5.3
	Other	7	1.7
In charge of food preparation	Mother	385	91.9
	Father	8	1.9
	Daughter	16	3.8
	Other	10	2.4
Head of the household	Mother	36	8.6
	Father	370	88.3
	Other	13	3.1
Occupation ^a^	Unemployed	49	11.7
	Skilled worker	105	25.1
	Employee	116	27.7
	Retired	126	30.1
	Professional	23	5.5
Education ^a^	Illiterate or primary school	94	22.4
	High school or diploma	229	54.7
	University degree	96	22.9
SES classes	Poor	130	31.0
	Middle class	172	41.1
	Rich	117	27.9

^a^ Variables belonging to the head of household.

**Table 3:** Factor-loading rotated matrix and Eigenvectors for the three identified dietary patterns.

Food Groups	Components		
	1 Unhealthy	2 Mediterranean	3 Traditional
Sugary beverages	0.679		
Salty snacks	0.676		
Mayonnaise	0.511		0.282
Sweets and desserts	0.480		
Refined grains	0.430		0.349
Red meat	0.414		
Hydrogenated fat with animal origin	0.396		
High-fat dairy products	0.389		
Processed meat	0.357		
Organ meat			
Olive		0.552	
Cruciferous vegetables		0.552	
Green leafy vegetables		0.540	0.494
Nuts	0.247	0.505	
Fish	0.223	0.503	
Dried fruits	0.269	0.501	
Fruits and fruit juice		0.435	0.368
Coffee	0.330	0.372	
Whole grains		0.358	
Low-fat dairy products		0.335	0.267
Tea			
Other vegetables		0.408	0.632
Eggs			0.494
Legumes		0.202	0.479
Pickle			0.441
Poultry	0.246		0.395
Potato			0.387
Margarine			0.362
Oil			0.256
Diet coke			

**Table 4:** Means and standard deviations of DPs among different SES classes.

SES Classes	*n*	DP Score Values					
		Unhealthy		Mediterranean		Traditional	
		Mean	SD	Mean	SD	Mean	SD
Poor	127	−0.205 ^a^	0.709	−0.380 ^a^	0.741	0.109 ^a^	1.198
Middle-class	170	−0.038 ^a^	0.963	0.063 ^b^	1.120	0.021 ^ab^	0.930
Rich	115	0.282 ^b^	1.244	0.327 ^c^	0.930	−0.152 ^b^	0.836

Different superscript letters indicate a significant difference at the *p* = 0.05 among the means in each column. The group sizes are unequal. The harmonic mean of the group sizes was used. SES = socioeconomic status; *n* = number; DP = dietary pattern; SD = standard deviation.

**Table 5:** Multiple linear regression coefficients and significances for predicting the HBW amount.

Dependent Variables	DPs						SES	
	Unhealthy		Mediterranean		Traditional			
	*Coef*	*p-Value*	*Coef*	*p-Value*	*Coef*	*p-Value*	*Coef*	*p-Value*
BW amount	0.407	0.017	0.102	0.563	−0.145	0.386	−0.006	0.954
TBW amount	0.355	0.060	0.095	0.625	0.117	0.526	−0.052	0.641
NTBW amount	0.268	0.364	−0.166	0.587	−0.423	0.157	0.110	0.561

BW = bread waste; TBW = traditional bread waste; NTBW = non-traditional bread waste; DP = dietary pattern; SES = socioeconomic status; *Coef* = coefficient.

**Table 6:** Binary logistic regression weights and significances for predicting NTB consumption.

Dependent Variables	DPs						SES	
	Unhealthy		Mediterranean		Traditional			
	*Expo Coef*	*p-Value*	*Expo Coef*	*p-Value*	*Expo Coef*	*p-Value*	*Expo Coef*	*p-Value*
NTB consumption	1.793	0.001	1.496	0.010	0.914	0.432	1.014	0.845

NTB = non-traditional bread; DP = dietary pattern; SES = socioeconomic status; *Expo Coef* = exponential coefficient.

**Table 7:** Binary logistic regression weights and significances for predicting bread wastage occurrence.

Dependent Variables	DPs						SES	
	Unhealthy		Mediterranean		Traditional			
	*Expo Coef*	*p-Value*	*Expo Coef*	*p-Value*	*Expo Coef*	*p-Value*	*Expo Coef*	*p-Value*
Bread WO	1.256	0.046	1.114	0.329	1.015	0.887	1.145	0.028
TB WO	1.168	0.142	1.050	0.649	1.046	0.660	1.113	0.082
NTB WO	1.211	0.103	1.025	0.841	0.904	0.433	1.232	0.008

WO = wastage occurrence; TB = traditional bread; NTB = non-traditional bread; DP = dietary pattern; SES = socioeconomic status; *Expo Coef* = exponential coefficient.

## Data Availability

The data presented in this study are available on request from the corresponding author. The data are not publicly available due to privacy restrictions.
